# So You Want to Teach a Field-Based Course: A Practical Toolkit for Teaching Teams

**DOI:** 10.1093/iob/obag047

**Published:** 2026-08-26

**Authors:** L M Arcila Hernández, A R Payne, N J Kaplanis, A L Borker, R R Dunkin, L M Alissa, L Balachandran, H A Bhatti, M Carr, D A Croll, G Dayton, G S Gilbert, E A Howard, P Kouba, K Kroeker, C Lay, H Leonard, A Martínez, R Mehta, T J Miller, I M Parker, M Pfau, E S Zavaleta, R S Beltran

**Affiliations:** Department of Ecology and Evolutionary Biology, University of California–Santa Cruz, Santa Cruz, CA, 95060 United States; Department of Ecology and Evolutionary Biology, University of California–Santa Cruz, Santa Cruz, CA, 95060 United States; Department of Ecology and Evolutionary Biology, University of California–Santa Cruz, Santa Cruz, CA, 95060 United States; Department of Ecology and Evolutionary Biology, University of California–Santa Cruz, Santa Cruz, CA, 95060 United States; Department of Ecology and Evolutionary Biology, University of California–Santa Cruz, Santa Cruz, CA, 95060 United States; Department of Ecology and Evolutionary Biology, University of California–Santa Cruz, Santa Cruz, CA, 95060 United States; Department of Ecology and Evolutionary Biology, University of California–Santa Cruz, Santa Cruz, CA, 95060 United States; Department of Ecology and Evolutionary Biology, University of California–Santa Cruz, Santa Cruz, CA, 95060 United States; Department of Biology, University of Massachusetts Boston, MA, USA; Department of Ecology and Evolutionary Biology, University of California–Santa Cruz, Santa Cruz, CA, 95060 United States; Department of Ecology and Evolutionary Biology, University of California–Santa Cruz, Santa Cruz, CA, 95060 United States; Department of Ecology and Evolutionary Biology, University of California–Santa Cruz, Santa Cruz, CA, 95060 United States; Department of Ecology and Evolutionary Biology, University of California–Santa Cruz, Santa Cruz, CA, 95060 United States; Environmental Studies Department, University of California-Santa Cruz, CA, 95060, United States; Department of Ecology and Evolutionary Biology, University of California–Santa Cruz, Santa Cruz, CA, 95060 United States; Younger Lagoon Reserve, University of California-Santa Cruz, CA, 95060, United States; Department of Ecology and Evolutionary Biology, University of California–Santa Cruz, Santa Cruz, CA, 95060 United States; Forestry and Environmental Management Department, University of New Brunswick, Fredericton, NB, E3B 5A3, Canada; Department of Ecology and Evolutionary Biology, University of California–Santa Cruz, Santa Cruz, CA, 95060 United States; Department of Ecology and Evolutionary Biology, University of California–Santa Cruz, Santa Cruz, CA, 95060 United States; Environmental Studies Department and Kenneth S. Norris center for Natural History, University of California-Santa Cruz, CA, 95060, United States; Department of Ecology and Evolutionary Biology, University of California–Santa Cruz, Santa Cruz, CA, 95060 United States; Department of Ecology and Evolutionary Biology, University of California–Santa Cruz, Santa Cruz, CA, 95060 United States; Department of Ecology and Evolutionary Biology, University of California–Santa Cruz, Santa Cruz, CA, 95060 United States; Department of Ecology and Evolutionary Biology, University of California–Santa Cruz, Santa Cruz, CA, 95060 United States; Department of Ecology and Evolutionary Biology, University of California–Santa Cruz, Santa Cruz, CA, 95060 United States; Department of Ecology and Evolutionary Biology, University of California–Santa Cruz, Santa Cruz, CA, 95060 United States; Department of Ecology and Evolutionary Biology, University of California–Santa Cruz, Santa Cruz, CA, 95060 United States; Department of Ecology and Evolutionary Biology, University of California–Santa Cruz, Santa Cruz, CA, 95060 United States

## Abstract

Field-based courses can be effective interventions in higher education but pose unique challenges. Instructors and teaching assistants (teaching teams) of field-based courses can use evidence-based course design to recruit, retain, and renew interest in life science fields while maximizing positive student experiences and outcomes. Yet, instructors and teaching assistants designing field-based courses in the life sciences lack an accessible, practitioner-focused synthesis of critical practices and tools for effective entry into this specialized form of teaching. We collected information about field-based teaching practices from educators at a four-year undergraduate research institution and hosted a collaborative summit with discussions focused around participants' lived experiences with field-based teaching to synthesize emergent themes. Additionally, summit moderators and participants acknowledged the importance of evidence-based teaching and pedagogical practices in field-based teaching. Because field-based courses require specialized teaching and leadership skills, we concluded that teaching teams should be trained in five critical practices of intentional course design and preparation: 1) providing context, 2) emphasizing community well-being, 3) preparing logistics, 4) developing interpersonal & communication skills, and 5) applying inclusive pedagogy strategies. Here, we discuss the use of these 5 critical practices in course design and we provide 10 inclusive pedagogical tools that can support positive student outcomes in field-based courses, including community building and support for project development and completion. We also emphasize the importance of support and teaching training for teaching teams of field-based experiences to improve student learning and community building outcomes in life science education.

## Introduction

Field-based courses can be transformative for students in the life sciences and other related fields (see transformative learning theory in Kitchenham 2008). These courses can uniquely benefit students through offering authentic opportunities to practice the scientific method in a natural setting and help build the practical and social skills needed to be a field scientist (Mogk and Goodwin 2012; Fleischner et al. 2013; Petcovic et al. 2014; Esparza and Smith 2023); see authentic learning environments in Herrington et al. (2014). Beyond providing important professional experiences for students, field-based courses can advance student outcomes and drive affective and behavioral outcomes known to propel science careers (Beltran et al. 2020; Shinbrot et al. 2022; Treibergs et al. 2022; Arcila Hernández et al. 2023; Esparza and Smith 2023; White et al. 2025). For instance, field-based courses can bring students, regardless of background or experience, to similar levels of field-specific knowledge (Mogk and Goodwin 2012; Beltran et al. 2020; Fairchild et al. 2024), affective outcomes (e.g., sense of belonging, self-efficacy), and behavioral outcomes (e.g., deciding to pursue a life science career) (Shinbrot et al. 2022; Treibergs et al. 2022; Esparza and Smith 2023; Fairchild et al. 2024; White et al. 2025). Particularly for field-based courses in biology, participants have reported a deeper connection to the natural world and the organisms they study (Treibergs et al. 2022; Arcila Hernández et al. 2023). Additionally, field-based courses can bring positive outcomes to instructors, who often find these courses exceptionally rewarding to teach because they increase mentoring opportunities and research collaborations (Hart et al. 2011; Fleischner et al. 2013; Petcovic et al. 2014).

However, field courses do not automatically work for every student. Costs, family or job obligations, unequal previous opportunities, lack of knowledge of hidden curriculum, structural barriers for students from historically marginalized identities, and other systematic barriers can preclude students from taking a field-based course or from having a beneficial experience while participating in one (Morales et al. 2020; Zavaleta et al. 2020; Peasland et al. 2021; Race et al. 2021), highlighting the need for intentional field-based course design (e.g., Morales et al. 2020; McGill et al. 2021; Alexander et al. 2024; Fairchild et al. 2024). Additionally, instructors of field-based experiences can be overwhelmed with pedagogical strategies, logistics, and safety concerns associated with traveling outdoors (Demarest 2014a; Boyle et al. 2007; Fleischner et al. 2017; O’Connell et al. 2021; Colon and Bayne 2026). Whether leading a field course or incorporating field-based activities into courses requires passionate, creative and experienced instructors who can apply thoughtful course designs outdoors (Boyle et al. 2007; Demarest 2014b; Giamellaro 2014; Yemini et al. 2025). However, effective application of pedagogical tools is not guaranteed in field-based courses because teaching teams often base their course design on anecdotal and personal experiences, using approaches that often reflect their own educational journeys. More recent, guiding frameworks for field course design became available, delineating relevant elements of field-based experiences that can help mitigate negative outcomes (Zavaleta et al. 2020; Race et al. 2021; O’Connell et al. 2022). There is also extensive research on place-based education, an overlapping teaching practice that emphasizes connection to local context and community (Demarest 2014b; Giamellaro 2014; Yemini et al. 2025). Specifically for the geosciences, a primer of place-based education was developed to summarize why and how place-based education can have an important role in undergraduate education (Semken et al. 2017). However, there is not yet an introductory practical guide of the critical practices and pedagogical approaches best suited for biology field-based courses in higher education.

We provide an introductory field-based teaching repository informed by the experiences of field-based instructors, and by a synthesis of evidence-based practices that highlight emerging critical practices and pedagogical tools identified during a field-based teaching summit. Our departments’ field-focused curriculum at a higher education institution provides a unique opportunity to gather common and important practices across a diverse set of field-based course instructors. For context, field-based teaching summit participants are instructors or education researchers of field-based courses in the life sciences, representing individuals at career stages ranging from graduate students to postdoctoral researchers to emeritus professors and staff members. Through the field-based teaching summit, we worked toward filling the aforementioned gap by providing a practical toolkit for field-based instruction and addressing the questions: What are the key characteristics of field-based courses at a higher education institution? What types of critical practices are used within field-based courses? How can we train instructors and teaching assistants to effectively implement best practices in field-based courses? Answering these questions is a critical next step toward closing the knowledge-practice gap in field-based teaching (Beltran et al. 2024).

## Reflections and synthesis procedures

### Positionality statement

All authors of this paper are affiliated with the University of California, Santa Cruz (UCSC), a Research 1 Minority-Serving institution. The Department of Ecology and Evolutionary Biology (EEB) provides a wealth of teaching experience that positions us well to summarize teaching tools in field-based courses. UCSC-EEB currently offers about 20 field-based courses per academic year (Supplemental Information: Table S1). Course variety is made possible through access to the University of California Natural Reserve System and the UCSC Campus Natural Reserve. Although all authors worked in the local context of UCSC, many have taught and experienced field-based courses at other higher education institutions. Many faculty in this department also prioritize offering experiential learning by engaging in education research that informs curricular decisions and seeking financial support to incorporate field-based activities into courses. We are a group of ecologists, conservation biologists, Natural Reserve staff, naturalists, and science educators at different career stages with a focus on field-based education research. Several of us identify with at least one historically marginalized identity group based on race and ethnicity, gender identity, or first-in-family [to attend college] status. We have all participated in or have taught an experiential learning program. As a result, we were uniquely poised to consolidate the extensive field experience of this community to provide a comprehensive toolkit.

### Reflections and synthesis

To gather and synthesize perspectives from our pool of field-based educators, we used several approaches. We first developed a survey to gather information about participants' teaching experience and practices. This survey presented key pedagogical strategies from the literature and asked practitioners about their familiarity and use of them, or if they use additional practices. We then held a field-based teaching summit to discuss the emergent themes based on the survey responses and to synthesize the most common practices and pedagogical tools. We also evaluated which practices are supported by evidence-based education research and identified which need further research support to demonstrate their effectiveness. Finally, to embed the synthesized knowledge within discipline-based education research frameworks, we then gathered additional key citations from the literature. From this iterative process we developed a set of critical practices and tools.

### Survey

Our implementation team (Authors: LMAH, ARP, NJK, ALB, ESZ, RSB, RRD) developed a survey to gather information on the types of field-based courses the participants had taught, and the type of pedagogical practices the participants used (Supplemental Information: Panel S1). We included summit participants as co-authors of this paper. Questions 1–19 were designed to capture the landscape of field-based experiences offered in the department and were based on relevant elements of field-based courses outlined in previous literature (Fleischner et al. 2013; O’Connell et al. 2021, 2022; Race et al. 2021; Shinbrot et al. 2022). The next two sections (Questions 20–27) specifically asked instructors about their teaching practices. The implementation team grounded these survey questions in evidence-based teaching practices known to benefit students that are relevant to field-based courses (Freeman et al. 2014; Sabel 2020; McGill et al. 2021; O’Connell et al. 2021, 2022; Race et al. 2021; Shortlidge et al. 2021; Shinbrot et al. 2022; Treibergs et al. 2022). The final three questions (Questions 28–30) asked about teaching training opportunities, importance, and challenges. The survey was given to educators from EEB and Environmental Studies departments that intended to participate in a field-based teaching summit, including the implementation team. We received 22 survey responses (out of 33 invitations), and summarized characteristics of field-based teaching courses, teaching experience, course evaluation methods, and teaching training of respondents. We used the survey as a tool to quantitatively summarize our thoughts and thus did not seek Institutional Review Board approval for our survey. One co-author, LMAH, analyzed and summarized the survey responses. For Likert scale questions, the data were presented using frequency distributions. For open ended questions relevant to teaching practices, the responses were read several times and added open codes as needed. Finally, the codes were grouped into emergent themes following Saldaña (2021). These emergent themes were discussed with the summit facilitators (ARP, NJK), who had read all the responses to discuss any potential disagreements in interpretation. Course enrollment data and analysis was approved following the protocol outlined in IRB #: HS-FY2025-12.

### Field-based teaching summit

The implementation team used survey responses to guide a 4-hour workshop to review field-based teaching practices (Arcila Hernández et al. 2026). The Likert scale question listing evidence-based teaching practices (Question 26) and the emergent themes on teaching practices were used to discuss which of these practices were most effective supporting learning outcomes through a gallery-walk activity. We included 9 options in the gallery-walk: scaffolding, peer-mentoring, iterative projects, observation-questioning-sampling, immersive field-experiences, low-stake projects, collaborative, structured community building activities, and unstructured community building activities. During the gallery-walk activity, participants provided examples and discussion points for each of these practices. We also discussed as a group any missing practices or any needed reframing of a practice. Finally, we asked participants, “What are the critical themes of field-based teaching practices?” Using a fishbone diagram, we synthesized participants’ responses to identify key themes that should be incorporated into a future field-teaching training program (Supplemental Information: Panel S2). While the summit emphasized participants’ lived experiences and did not provide an initial educational framework for discussion, several of the participants were familiar with and practiced evidence-based and inclusive teaching practices, including at least 7 participants who have published manuscripts on biology-based education research. Therefore, reflections and discussions were often based on teaching practices supported by the education research literature. We acknowledge that our discussions are biased towards the context of one educational institution, and in an effort to provide a wider context, we supply here a brief review of which of these practices need further support of their effectiveness in the literature and which ones are established evidence-based teaching practices. We also reviewed the critical themes and pedagogical tools below by complimenting information from the literature on field-based learning, but specially, we relied on publications from two previous research groups studying field-based education: the Undergraduate Field Experience Research Network (U-FERN. O’Connell 2018) and the Natural History Institute’s guide “Saying Yes to Environmental Field Studies: A Guide to Proactive, Successful Administration and Operations” (Pace et al. 2017). This manuscript was developed following the summit.

## Summit summary

### Participants

Most survey respondents had been either an instructor (68%) or a teaching assistant (18%) of a field-based experience. Respondents with experience in field-based teaching taught a total of 19 different field-based courses (a subset of Supplemental Information: Table S1). Over 50% of respondents taught a field-based course at least once per year.

### Characteristics of the field-based courses

**Fig. 1:**
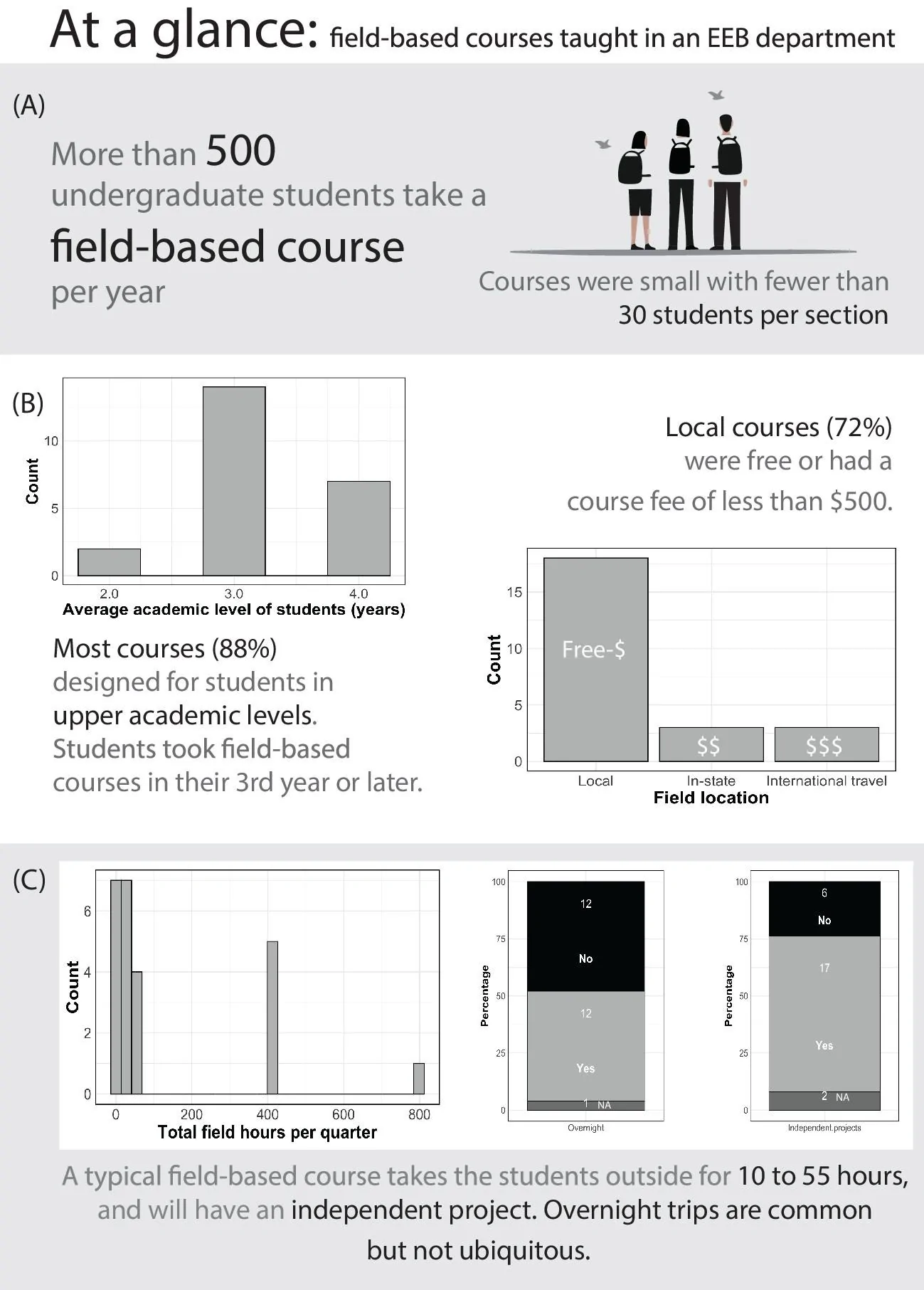
Characteristics of field-based courses in EEB and two other field-based courses in an adjacent department, most of them taught by participants of the Field-based Teaching Summit at the University of California, Santa Cruz. This figure summarizes Supplemental Information: Table S1. We show a general review of student enrollments in the field-based courses (panel A), followed by a summary of the academic level of students (calculated by determining when in their time at the university the students took the course, as a percentage). We then back-calculated to standardize for a 4-year degree program), course location, and fees (panel B). We also show a few relevant elements of these field-based courses (panel C), such as number of hours in the field, presence of overnight trips, and presence of independent projects. Figure created by L. Arcila Hernández modifying generative AI icons of student groups from Adobe Illustrator 2025.

The type of field teaching included a wide range of courses. Field course immersion varied from short (i.e., a few hours, or half-day) local field trips to intensive weeks-long courses with substantial travel (Figure 1). Instructors brought students to a variety of field locations: 16 courses took place in local natural environments (within 50 miles of campus), 3 took place farther but stayed within California, and 3 took place internationally (Figure 1-B). Field visits ranged from once per week (56%) to more regular immersive experiences (every day) (31%). Our survey data showed that most courses had a small class size (15–22 students per section, with an average of 8.6 students per instructor). Course learning outcomes emphasized field research (e.g., improving understanding of natural history, ecology, and evolutionary process), professional development (e.g., building scientific and field research skills), and community building outcomes (e.g., building community among students).

### Critical practices

**Fig. 2:**
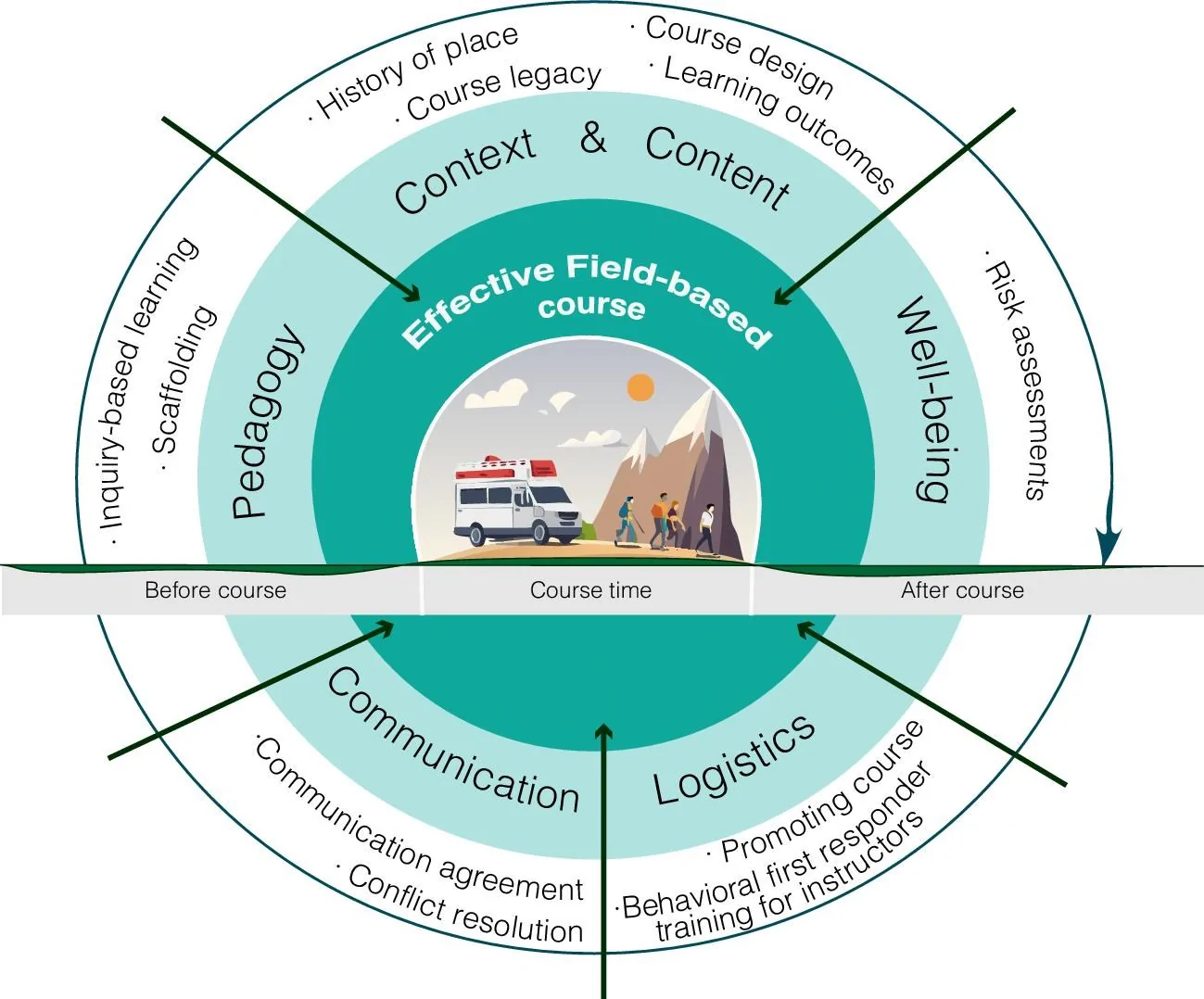
Critical practices (middle circle) influence a field-based course during and outside of course time (inner circle). Inclusive pedagogical tools (outer circle) can be used at every stage of a field-based course development and implementation in an iterative process (green arrow around outer circle). Here, we provide a few examples of inclusive pedagogical tools that can be used under each of the critical practices. Figure created by L. Arcila Hernández modifying generative AI icons of student groups, vans, and the outdoors from Adobe Illustrator 2025.

From our survey and summit discussions, we distilled the most important considerations for current and potential instructors of field-based courses (Figure 2). These considerations reflect recurrent practices that were present in field-based courses that aim to maximize students’ cognitive, affective, and behavioral outcomes (Beltran et al. 2020; Race et al. 2021; Arcila Hernández et al. 2026). While the survey was developed with evidence-based practices in mind, the additional practices from the summit emerged from the lived experiences of the summit participants and reflect the lessons learned of this community. Our goal with this section is to provide a starting point for instructors to begin exploration of these practices and how to incorporate them into their particular learning experiences. Wherever relevant citations are available, we provide them to explicitly link our suggested practices to relevant theoretical frameworks, and to allow interested practitioners to develop a deeper understanding of each practice.

1. ***Provide context & content.*** At the beginning of a field-based experience, lead instructors should discuss course content, desired educational outcomes, pedagogical approach, and anticipated student needs with their teaching teams (if there is a teaching team; we recognize that many instructors lead these experiences on their own). Course materials and protocols should be revised to ensure they align with the goals of the course. Course materials and introductory content should also be provided to the teaching team and students, including course learning goals, motivations, values, expectations, and what is new in the course. Students, co-instructors, and teaching assistants need to understand the course context in order to orient to the environment in which learning is to take place, build trust, support curricular decisions, and foster cultural understanding and respect.
  For instance, to encourage a sense of belonging and appreciation among the students, it is critical to acknowledge the history of the locations that will be visited, as well as the relationships and impacts that humans have with that place (Rinkevich et al. 2011; Fleischner et al. 2017; Greene 2017; Semken et al. 2017). We encourage instructors to think broadly about how identity and cultural context impact the students' experience. We know from culturally responsive pedagogy and place-based education that students gain in knowledge and affective outcomes when the course acknowledges Indigenous land history, local community dynamics, and environmental justice issues anchoring the biology in a critical, real-world context (Demarest 2014b; Ladson-Billings 1995; Hammond and Jackson 2015). The literature on these topics is rich and growing, and Cotton (Cotton 2009), Giamellaro (2014), and Semken et al (2017), provide more about novelty space, context of place, and how to incorporate important context into field-based experiences. The teaching team should also provide students with background information on the course location(s), introductory physical and biological natural history, available resources (e.g., infrastructure such as vans, field gear libraries, etc), and mentoring opportunities. Clear communication around context can facilitate cultural understanding and respect between all participants.
2. ***Build a course culture of well-being*.** Field-based courses can carry greater physical and mental health risks than typical courses. To mitigate these challenges, instructors should cultivate a course culture of safety and well-being from day one, emphasizing how everyone can play a role in minimizing risks and having a positive experience. Embedding these practices early allows the students to transform into a real community of practice (Wenger 1998). Within this framework, students shift from passive learners to active collaborators, supporting one another as they navigate field risks and co-construct knowledge of local flora and fauna. Instructors should cultivate an environment that actively invites safety inquiries, while remaining cognizant that power dynamics and past experiences may impact a student’s willingness to disclose concerns (Carello and Butler 2015). Instructors should also make sure that essential safety equipment is available (e.g., satellite phones and life-saving equipment such as Epipens) and have training on how to manage risks and respond to incidents. A site-specific analysis of risks particular to the group and field location should be developed into a field safety plan (Demery and Pipkin 2021). See Supplemental Information: Panel S1). Plans should include emergency medical and communication protocols, maps, and essential safety equipment needs. These plans are crucial risk mitigation tools that should be provided and discussed with appropriate campus administrators and the teaching team, with key aspects communicated with the students prior to field departure. For detailed guides on risk management, first aid training requirements, and emergency communication protocols, we encourage instructors to read (Pace et al. 2017; Demery and Pipkin 2021). Instructors should also anticipate organism-specific hazards (e.g., venomous species, disease vectors, marine stingers) and use natural history knowledge to help students understand these organisms beyond the threat they pose. Building a course culture of well-being should also include measures to support mental health (John and Khan 2018; Eifling 2021) and accessibility (Ramírez-Castañeda et al. 2022), as well as proactive measures to prevent sexual harassment, sexual assault, and other forms of aggression (Cronin et al. 2024). Everyone should know where to access the field safety plan, equipment, and other well-being protocols.
3. ***Prepare logistics: before, during, and after course*.** A considerable portion of a field-based instructor’s time would necessarily be used on logistics to assure that the course runs smoothly, including recruiting and selecting participants, transportation and accommodation, food requirements, tracking, and storage of essential equipment (e.g., tents, sleeping bags, research equipment) (Fleischner et al. 2017). We provide a checklist with some of the most common items that need to be in place to run a field-based course in the supplemental information (Panel S3). For further guidance on logistics and procedures in field settings, we recommend Pace et al. (2017, sections XI–XIII) and the discussion of local learning challenges in Demarest (2014b, Part III).
  Here, we want to highlight the importance of a student recruitment plan and organizational meetings, which is not often described elsewhere. Many students in the life sciences are not aware of field-based courses or the opportunities they bring to their careers (Giroux and Purpel 1983; Shinbrot et al. 2022; Peasland et al. 2024). Whether there is an application process for the course or not, instructors should proactively plan how, when, and where they will recruit students or promote the course, assuring that students from all backgrounds know about the course and how it can benefit them.
  A pre-course meeting for the teaching team helps everyone understand the course priorities, expectations, and timelines. Similarly, a pre-course meeting for the students is needed to introduce expectations, provide required/suggested personal and travelling checklists, discuss accommodations, special food needs and allergies, provide relevant information on financial aid and course credits, guidelines, and answer any questions the students might have about the field-based experience. Learning about this context also decreases the cognitive load that the students might experience while having their first immersive experience, allowing them to have more opportunities to learn about local natural history (Sweller 1988, 2011). Instructors should also be prepared to make decisions about transportation, food, lodging, pacing, and have field-specific knowledge, as well as knowledge about infrastructure of sites.
4. ***Develop interpersonal and communication skills*.** Field-based courses offer a unique combination of formal and informal settings that foster the development of students' interpersonal and communication skills. Formal settings include collaborative inquiry-based projects and informal settings often revolve around cooperative activities, including menu planning and setting up camps (Mogk and Goodwin 2012). These interactions nurture teamwork, leadership, accountability, and conflict-resolution skills (Demarest 2014b; Boyle et al. 2007; Fedesco et al. 2020; Arcila Hernández et al. 2023). Additionally, informal engagements can “demystify” teaching teams and empower the students to join them in exploration as near-equal partners (Streule and Craig 2016).
  Instructors can establish clear expectations and boundaries around communication through community agreements, consensus-building, or similar approaches (see Supplemental Information: Panel S3). Communication agreements should include principles such as respect, self-advocacy, timely responsiveness, conflict resolution, and creating opportunities for all voices to be heard. Equally important is clearly communicating unacceptable behaviors (e.g., sexual or racial harassment, violent language) within the framework of institutional policies (Cronin et al. 2024), explaining why these behaviors are unacceptable, and clearly detailing the consequences of violating course expectations. Teaching teams play a crucial role by modeling these communication standards consistently and positively.
5. ***Prioritize inclusive pedagogy.*** Across all critical practices, it is paramount to intentionally choose pedagogical tools that are student-centered supporting students’ growth, not only academically, but also other behavioral and attitudinal endeavors (Demarest 2014b; Morales et al. 2020; O’Connell et al. 2022). In our summit, we identified 10 pedagogical tools that can help field-based instructors create positive outcomes for students, all of which are already evidence-based best practices inside classrooms. Below, we argue why and how these pedagogical tools can be successfully translated to field-based courses, with relevant definitions, references, and examples.

### Pedagogical tools

To address our final research question on how to train teaching teams to employ these best practices in field-based courses, we identified 10 pedagogical tools from the education literature and instructors' lived-experiences that can positively transform field-based courses (see Table 1 for definitions and examples of each tool). First, instructors should develop content and activities using *(1) backwards design*, keeping learning outcomes and objectives in mind (Wiggins and McTighe 2001). For field-based courses, instructors can make use of the Undergraduate Field Experiences Research Network (UFERN) model (O’Connell 2018, 2022) and the persistence in a biological science major framework (Race et al. 2021) to design the main elements of their course. Inclusive and equity-minded designs can also be incorporated during course development (e.g., Morales et al. 2020; Zavaleta et al. 2020).

**Table 1:** Inclusive pedagogical tools that enhance field-based learning in the life sciences.

**Pedagogical tool**	Summit Definition*	**Canonical definition (Relevant Citations)**	**Evidence-based support**	**Examples in field-based experiences**
**Backwards design**	Designing a course with learning outcomes and objectives in mind	Structures curriculum by first identifying desired learning outcomes, determining acceptable assessments, and finally planning specific learning experiences and instruction. (Biggs 1996; Wiggins and McTighe 2001; Ambrose et al. 2010; Fink 2013; Freeman et al. 2014)	Yes, strong evidence for improved alignment of course features and learning outcomes. Evidence within classroom and laboratory settings.	• Planning rapid research projects around the learning outcome of “Implementing the scientific method”
**Scaffolding**	Instructors successively build challenges as students gain skills and confidence.	Provides temporary, adaptive support structures to assist students as they develop new skills or concepts, which are systematically tapered off as the learner gains independence. (Wood et al. 1976; Vygotsky 1978; Reiser 2004; Wilson 2012; Sabel 2020)	Yes, extensive empirical support for cognitive gains in students when using scaffolding. Evidence within classroom and laboratory settings.	• Begin with the entire class collecting and analyzing data before moving on to individual projects • Moving from 15-minute projects to week-long projects • Practice methods before moving on to research design
**Peer-mentoring**	When people of similar experience levels work together to help each other learn and grow.	A reciprocal, collaborative learning relationship where individuals of similar developmental or experience levels (*peers*) provide psychosocial, academic, or technical support to one another. (Topping 1998; Nora and Crisp 2007; Crisp and Cruz 2009; Sacerdote 2014; Wilton et al. 2021; Esparza and Smith 2023)	Yes, support for peer-mentoring impacting students academic persistence, belonging, and skill development. Evidence within classroom, laboratory, and field-based settings.	• Think, Pair, Share activities • Group work throughout a course, with groups mixed up across the course • Having former students visit, mentor, guest lecture, or hired as teaching staff
**Iterative projects**	Learning through cyclical inquiry, enabling multiple opportunities to revisit and reflect on ideas or practice skills.	Based on cyclical inquiry, where students repetitively engage with a task, concept, or skill, using formative feedback to continuously revise and deepen their conceptual understanding. (Blumenfeld et al. 1991; Nicol and Macfarlane-Dick 2006; Hattie and Timperley 2007; Aguilera et al. 2017; Lang 2021; Race et al. 2021)	Yes, support for iterative, repeated instruction improving learning. Evidence within classroom, laboratory, and field-based settings.	• Revision based on peer review • Case studies built on top of basic skills • Course-long projects that require continued research and feedback • Repeating a project
**Inquiry-led learning**	Engaging students through real-world exploration and questioning.	Instruction is driven by student-generated questions, problem-solving, and investigation, shifting the instructor’s role from a primary information source to a facilitator. (Prince and Felder 2006; Lee 2012; Mogk and Goodwin 2012; Freeman et al. 2014; Attard et al. 2021)	Yes, extensive evidence across STEM education research. Evidence within classroom, laboratory, and field-based settings.	• An exercise where students generate and share questions • Instructor modeling hypothesis generation • Activities that build on observations and lead to questions
**Immersive learning**	Opportunities to see, touch, and interact with the subject matter and observe the wider context.	Deeply embeds learners within a specific physical, ecological, or socio-cultural environment, leveraging direct sensory interaction and real-world context to construct knowledge. (Kolb 1984; Lave and Wenger 1991; Mead et al. 2019; Shaulskiy et al. 2022; Saha et al. 2024)	Yes, moderate-to-strong evidence. Evidence within classroom, laboratory, and field-based settings.	• Detailed, independent field journals with guidance on how to make observations (engage senses, mindfulness, reflections, etc) • Observe an organism/environmental system for a long period of time, or return to a single spot over several days to observe changes.
**Low-stakes projects**	Evaluations that don’t heavily impact final grades, but help students gain skills.	Formative, low-consequence assessments designed to provide frequent feedback and practice opportunities. (Black and Wiliam 1998; Nicol and Macfarlane-Dick 2006; RoedigerIII and Karpicke 2006; Rivera et al. 2024; White et al. 2025)	Yes, strong support for low stakes projects helping reduce student anxiety and improve retention. Evidence within classroom, laboratory, and field-based settings.	• Projects where students complete the entire project in one session • Formative assessments
**Collaborative learning**	When students work together to solve complex problems.	Students work in structured groups to co-construct knowledge, solve complex tasks, or achieve shared intellectual goals through mutual accountability and social interaction. (Slavin 1996; Prince 2004; Freeman et al. 2014; Scager et al. 2016; Ramdani et al. 2022)	Yes, extensive evidence of positive student outcomes in classrooms. Evidence within classroom and laboratory settings.	• Rapid research projects in small groups • Co-working and providing feedback
**Structured community building**	Explicit, required opportunities for building relationships, often initiated by the instructor.	Deliberate, instructor-mediated interventions designed to cultivate social cohesion, and an explicit sense of academic and interpersonal belonging and safety among participants. (Tinto 1997; Hurtado et al. 1999; Michaelsen and Sweet 2008; Donovan et al. 2018; Strayhorn 2018)	Yes, growing evidence linking student belonging to increased retention and achievement. Evidence within classroom, laboratory, and field-based settings.	• Daily group check-ins • Facilitated group interactions with randomization of members and roles-ensuring that everyone participates • Groups to help with daily chores and cooking
**Unstructured community building**	Opportunities to build relationships outside the official curriculum.	Co-curricular or informal socialization opportunities occurring outside the formal syllabus that facilitate organic relationship building, student integration, and peer capital. (Lave and Wenger 1991; Tinto 1997; Astin 1997; Stassen 2003; Feldman and Matjasko 2005; Kuh 2008; Conradson 2016)	Some support, less formal evidence. Some evidence outside and within classrooms but not in field-based settings.	• Campfires, games, and music • Free time to get to know each other • Mixing up informal opportunities like who rides in which van

*Notes*: *We provide definitions used during the summit discussions, as they reflect instructors' understanding of the pedagogical tools and not necessarily a broader understanding of the topic in the educational literature as shown in the canonical definitions and relevant citations.

Instructors can use a combination of *(2) scaffolding* and *(3) iterative projects* to provide students with opportunities to learn new skills and practice them (e.g., measuring tree growth using the diameter at breast height (dbh) measurement, first practicing alongside an instructor with the campus trees and then a group of students work together to measure trees in a long standing forest quadrant (Kolb 1984; Reiser 2004; Aguilera et al. 2017). While scaffolding assures that students are learning skills in a progressive and guided manner, iterative projects, paired with *(4) low-stakes projects*, give students the opportunity to practice those skills in an environment where making a mistake is safe and will not have a big impact on their grades (Sambell and Hubbard 2004; Rivera et al. 2024). Overall, these practices aim to increase scientific skills and self-confidence in the students.

**Fig. 3:**
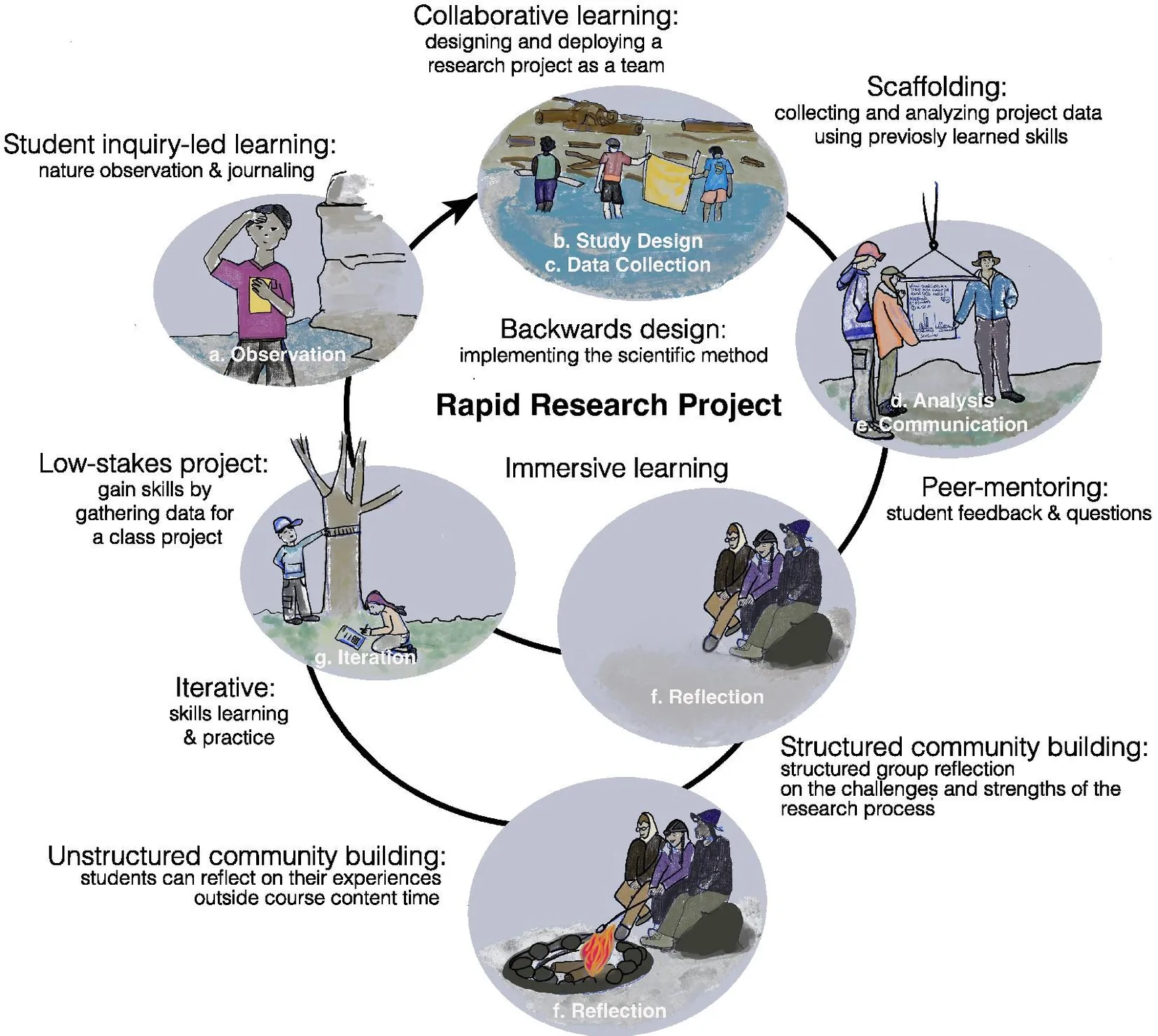
Implementation of all 10 pedagogical practices in a rapid research project where the students (a) observe patterns in nature and generate questions; then (b) collaborative design and implement an experiment in one day or less. After (c) data collection and (d) analysis, (e) students present their research to their peers or community members. The experience of performing science publicly (b-e) might be a critical component of forming science identity. (f) Reflection on the research challenges and strengths can happen both during structured and unstructured course activities. Figure created by L. Arcila Hernández using Procreate 2025 and Adobe Illustrator 2025.

Field based-experiences provide fantastic opportunities to engage in *(5) inquiry-led learning* and (6) *immersive learning* (Lee 2012; Fleischner et al. 2017; Shaulskiy et al. 2022; Saha et al. 2024). For example, Rapid Research Projects, which often take students to a new location, provide opportunities for students to observe organisms and generate questions about their environment, define a hypothesis, and develop a study, then analyze and interpret data through a presentation or write-up, usually in a single day (Figure 3). By engaging the students with student-led exploration and questioning of real-world issues, as well as providing space to be fully immersed in those questions and environments, students gain not only on affective outcomes (e.g., scientific identity) but also gain cognitive and scientific skills (Boyle et al. 2007; Shinbrot et al. 2022; Treibergs et al. 2022).

Finally, pedagogical approaches that harness the power of personal connections among the students should be implemented. Building these connections facilitates a student’s sense of belonging, science identity, a sense of purpose, and development of scientific skills such as communication and teamwork that are the foundation for collaboration in science; among many other affective and cognitive outcomes (Beltran et al. 2020; Peasland et al. 2021; Race et al. 2021; Shaulskiy et al. 2022; Arcila Hernández et al. 2023). These approaches include, but are not limited to, (7) *peer mentoring, (8) collaborative learning, (9) structured community building*, and *(10) unstructured community building* (Tinto 1997; Hurtado et al. 1999; Kuh 2008; Freeman et al. 2014; Shinbrot et al. 2022; Esparza and Smith 2023). Through *(7) peer mentoring* students can act as practitioners, developing self-efficacy. Through *(8) collaborative learning* students can work together toward common goals and develop intellectual, social, and communication skills while engaging with authentic practices. Field-based courses offer opportunities for both *(9) structured communit*y building facilitated by course activities and learning structures, and *(10) unstructured community building*, where informal interactions (e.g., campfire time, shared cooking) allow development of social connections (Fedesco et al. 2020, Morales et al. 2020, O’Connell et al. 2021, 2022).

## Discussion

We synthesized common practices and tools across varying field-based experiences to maximize positive student outcomes (Figure 2, Figure 3, and Table 1). The summit was an opportunity to gather extensive practice-based insight to better understand the participants' teaching experience. This process allowed us to evaluate whether evidence based practices are commonly employed, and to identify other practices that may need additional research regarding their effectiveness. While emerging independently from other frameworks, these critical practices align and complement previous discussions on how to design field-based experiences (Fleischner et al. 2013, 2017; Semken et al. 2017; O’Connell et al. 2022). We go beyond these prior individual frameworks to provide a practical introductory toolkit of inclusive pedagogical practices that teaching teams can use at every stage of development and implementation of their field-based experiences.

Field-based experiences are high-impact practices (Kuh 2008) that naturally align with inclusive pedagogical approaches (O’Connell et al. 2021; Arcila Hernández et al. 2024). In most cases, implementation of the approaches summarized here should be straightforward and do not need to start with a full-scale immersion course. For example, instructors could easily adapt all 10 pedagogical tools by facilitating Rapid Research Projects during a few hours at a local park (Figure 3). While inclusive consideration of diverse abilities is integral to each of the critical teaching practices, in-depth discussion of strategies for designing field-based experiences that recognize and support learners across a spectrum of abilities was minimal during the summit. Yet participants agreed that fostering accessible and supportive learning environments is a critical component of effective field instruction and noted the need for more focused discussion on this topic. Recent syntheses highlight that students with disabilities continue to encounter substantial barriers in field-based learning, underscoring the need for instructor preparation in accessible and inclusive field-course design (Stokes et al. 2019; Atchison et al. 2022; Chasen et al. 2025).

The field-based teaching summit was the first time many of us had the opportunity to share challenges and opportunities of field teaching in biology and conservation with other instructors and staff, highlighting the benefits of having a community of practice surrounding these topics at every institution. When discussing critical practices and pedagogical tools, it became clear that there are ample opportunities to collaborate in the development and assessment of those practices. For those interested in starting or continuing to assess their field-based experiences, the UFERN project already developed a comprehensive guide on tools and considerations needed to evaluate outcomes (Shortlidge et al. 2021). We agreed with previous work (Shortlidge et al. 2021; Shinbrot et al. 2022) on the need for additional research demonstrating the benefits (and limitations) of field-based learning in closing opportunity gaps, supporting longer-term career goals, inspiring students, and developing environmentally-concerned community members. Instructor collaboration might become especially relevant to build capacity for longitudinal assessments aiming to understand long-term outcomes of field-based learning.

We found that many instructors did not have training on any of the critical practices or inclusive pedagogical approaches necessary for field-based teaching at the start of their teaching careers and acquired them with time. Given the high impact of field-based experiences and the challenges associated with instruction (Mogk and Goodwin 2012; Shinbrot et al. 2022; Beltran et al. 2024), we call for institutions to provide explicit instruction, financial, and logistical support for teaching teams related to the critical practices outlined here and incentives for teaching field courses. A training, designed with critical practices and community building in mind, could build capacity for teaching field-based experiences. Building a community among instructors can help them engage with recurrent workshops for sharing field-based learning approaches, challenges, and solutions. We need to support instructors by offering skill sets centered on these critical practices while giving them a community to ask and answer questions about how to teach a field course. New instructors excited to take on the teaching of field-based courses should not be overburdened under financial and logistical constraints but instead supported in their endeavor to positively impact a new generation of scientists.

### Scope and transferability

While the five critical practices identified here should be universal principles of effective field-based learning, we recognize that our institutional context—a large research university in the Global North with established infrastructure—presents unique privileges. For this toolkit to serve as a scalable, cross-institutional framework, teaching teams should adapt it to their distinct institutional realities and structural barriers such as cost, liability, and land access. For instance, instructors at a smaller or resource-constrained institution with smaller class sizes but no vehicle fleet could leverage opportunities to develop community and sense of belonging through collaborative and place-based learning at public-transit accessible locations such as parks or campus green areas. Similarly, resource-constrained institutions may not be able to support multiweek field trips. Nonetheless, instructors can achieve meaningful outcomes using scaffolding and iterative projects during single-day trips to accessible natural or urban spaces. The pedagogical tools in Table 1 provide a flexible framework that institutions can adapt to their unique contexts and constraints while supporting positive student outcomes.

### Conclusion

Education research consistently demonstrates that field-based courses are transformative experiences, particularly when instructional design intentionally balances students' cognitive and affective needs with structured learning outcomes (Kitchenham 2008; O’Connell et al. 2022). However, there is a persistent gap between insights from biology education education research and daily instructor practices (Beltran et al. 2024). By establishing a community of practice at our institution, we brought together experienced instructors of field-based courses and biology education researchers to bridge this gap. We synthesized our department’s field-based courses and educational approaches to study organismal biology and conservation and contrasted them with current evidence-based educational frameworks. With this practitioner-informed synthesis, we provide a toolkit that field-based instructors can use to determine what are the critical practices of field-based teaching and how to put those into practice. Ultimately, while this toolkit offers actionable strategies for immediate implementation, we call on other institutions to initiate similar reflective, collaborative summits. Cultivating these communities of practice is vital to equipping teaching teams, breaking down systemic barriers in the field sciences, and training the next generation of integrative biologists.

## Supplementary Material

obag047_Supplemental_File

## Data Availability

Data and/or code will be accessible via the following link: Arcila Hernández, Lina; Payne, Allison R.; Kaplanis, Nikolas J.; Borker, Abraham L.; Dunkin, Robin R.; Alissa, Louise M.; et al. (2025). Characteristics of field-based and experiential courses available as of 2025 in two life science departments in a higher education institution. figshare. Dataset. https://doi.org/10.6084/m9.figshare.29564996
