# Supplementary material for: So You Want to Teach a Field-Based Course: A Practical Toolkit for Teaching Teams
Source: Integr Org Biol. 2026 Aug 26;8(1):obag047. doi: 10.1093/iob/obag047 (PMC13573308; doi:10.1093/iob/obag047)
Supplement: Supplementary file 1 [file obag047_supplemental_file.docx]

# So you want to teach a field-based course: a practical toolkit for teaching teams

# Supplemental Information

## Lina M. Arcila Hernández ([larcilah@ucsc.edu](mailto:larcilah@ucsc.edu)), Allison R. Payne, Nikolas J. Kaplanis, Abraham L. Borker, Robin R. Dunkin, Louise M. Alissa, Lalitha Balachandran, Haider A. Bhatti, Mark Carr, Don Croll, Gage Dayton, Gregory Gilbert, Elizabeth A. Howard, Paige Kouba, Kristy Kroeker, Chris Lay, Hugh Leonard, Ari Martínez, Rita Mehta, Timothy J Miller, Ingrid M. Parker, Madison Pfau, Erika S. Zavaleta, Roxanne S. Beltran.

**Table S1.**

See table in the following data repository:

Arcila Hernández, Lina; Payne, Allison R.; Kaplanis, Nikolas J.; Borker, Abraham L.; Dunkin, Robin R.; Alissa, Louise M.; et al. (2025). Characteristics of field-based and experiential courses available as of 2025 in two life science departments in a higher education institution. figshare. Dataset. <https://doi.org/10.6084/m9.figshare.29564996>

**Panel S1: survey**

Field-Based Teaching Practices Survey

Thank you for taking our survey on field-based teaching practices! This survey will provide the foundation for a manuscript that we will write together after the Summit. It should take approximately 20 to 25 minutes to complete (including your introductory slide). If you don't know an answer, it's okay to leave it blank.

**Field-Based Teaching Practices**

Please focus on subject area specific (i.e. EEB) undergraduate courses.

1. What field courses have you taught? e.g. BIOE 151ABCD Ecology & Conservation in Practice
2. How many times total have you taught field courses anywhere? (i.e., how many quarters/semesters)
3. How often do you teach field courses?
   i. *Mark only one oval.*
   ii. Rarely
   iii. Once every few years
   iv. Once a year
   v. Multiple classes a year
4. Describing current field-based courses and the teaching community
5. Please answer the following questions based on the field course you have spent the most time on.
6. What is/was your primary role in teaching this field course?
   i. *Check all that apply.*
   ii. Teaching assistant
   iii. Co-instructor
   iv. Lead instructor
7. How often is your field course offered?
   i. *Mark only one oval.*
   ii. Every other year
   iii. Every year
   iv. Every quarter
   v. Other:
8. What is the student/instructor ratio for your class (e.g. 28 students/3 instructors)? Would you make any changes to it? (Please include TAs as instructors.)
9. How many TAs do you have? Would you make any changes to that number?
10. How many students do you have per section?
    i. *Mark only one oval.*
    ii. 1 - 15
    iii. 15 - 22
    iv. 22-30
    v. 30+
    vi. 13.
11. How far does your field class travel?
    i. *Check all that apply.*
    ii. Locally (within 50 miles of Santa Cruz)
    iii. Statewide
    iv. Nationally
    v. Internationally
    vi. Other:
12. How many times, on average, do you go to the field in your field-based course?
    i. *Mark only one oval.*
    ii. Once a week
    iii. Twice a week
    iv. Three times a week
    v. Other:
13. What kinds of field experiences do your students get as a part of the course?
    i. *Check all that apply.*
    ii. 1-2 hours of outdoor time
    iii. Multiple day trips
    iv. 1 overnight trip
    v. Multiple overnight trips
    vi. Multi-week experiences
    vii. Other:
14. How much does it cost (total budget per quarter to you/the university) to put on your course beyond the typical instructor/TA salary? For example, renting vans, paying guest speakers, food costs, UC reserve fees, etc.
15. How much do students pay to participate in the course?
    i. *Mark only one oval.*
    ii. Nothing
    iii. $10-30
    iv. $30-100
    v. $100-500
    vi. $500+
    vii. Other:
16. Please describe any scholarships available for students to participate in the course.
17. How do you market your field course?
    i. *Check all that apply.*
    ii. Word-of-mouth
    iii. Course Catalogue
    iv. Department reaches out through emails
    v. Instructor reaches out through emails
    vi. Advertisement posters
    vii. EEB social media
    viii. EEB field course website
    ix. Other:
18. Do students need to apply to get into the class? Please describe any application/interview processes, as well as any selection criteria.
19. Please upload the syllabi for your field courses to this folder.

**Field-based teaching practices**

These questions are meant to describe practices applied across courses you have taught.

20. What are the most important learning outcomes you include in your field courses?
21. Did you do any specific activities to support those course learning outcomes?
22. For one of the learning outcomes listed above, describe one practice that you think is particularly effective for achieving that outcome.
23. What community building activities, if any, do you use in your field classes?
24. What are the common, openly recognized challenges of teaching field courses?
25. What are the overlooked challenges of teaching field courses?
26. Which of these common field-based practices do you think are most important to incorporate into your courses?
    i. *Mark only one oval per row.*
    ii. Not important | A little important | Somewhat important | Important | Very important
    iii. Field journals
    iv. Field observations
    v. Student-initiated research
    vi. Team- initiated research
    vii. Instructor- initiated research
    viii. Scaffolded projects(building throughout the quarter)
    ix. Iterative projects(repeated practice)
    x. Low-stakes projects
    xi. Student presentations
    xii. Team-work
    xiii. Collecting data for a long term data set

**Course Evaluation**

27. What tools do you use to evaluate the effectiveness of your course? (e.g. end-of-course surveys, SETS, students reflections, etc) Please describe.

**Field-based teaching training**

28. What do you think are some important components of a training that would help people teach field-based classes?
29. Have you received training focused on building equity, safety, and /or cultural responsibility into field-based courses? Please describe.
30. What are the most common training deficits for field-based instructors?

**Panel S2: workshop slides**

A redacted copy of the slide show used during the Field-based Teaching Summit held at the University of California Santa Cruz on 25 October, 2024 can be found here:

Arcila Hernández, L.; Beltran, R. S.; Zavaleta, E; Borker, A.L.; Dunkin, Robin R.; Payne, Allison R.; Kaplanis, N.J. (2026). Field-based teaching summit 2024: Workshop Slides. Figshare. Presentation. <https://doi.org/10.6084/m9.figshare.31416680>

**Panel S3: check list and resources**

1) **Field-based course check list**

**Critical Practices**

- ***Provide Context***
  - Educational outcomes
  - Course learning goals
  - Pedagogical approaches
  - Course expectations
  - Course content
  - Course protocol
  - History of place
  - Course history and any new changes to the course
- ***Build a course culture of well-being***
  - Activity to emphasize everyone's role in minimizing risks and having a positive experience
  - Acquire or determine if essential safety equipment is available
  - Develop a protocol for how the teaching team and students can raise safety concerns
  - Emergency medical protocol
  - Communication protocols
  - Maps
  - Measure to support mental-health
  - Measures to support accessibility
  - Measures to prevent sexual harassment, sexual assault, and other forms of aggression
  - Other safety equipment
- ***Prepare logistics***
  - Know your topic and refresh information on relevant topics.
  - Know your location and obtain the most up to date information on infrastructure
  - Actively recruit participants
  - Select participant if needed
  - Decide on transportation needs
  - Book vans, buses, or airplane tickets as needed
  - Decide and book accommodations
  - If camping
    - book camping locations
    - Have available camping equipment as needed
    - Tents
  - Sleeping bag
  - Sleeping pads
  - Headlights or other lights
  - Buy or make sure that sanitary food preparation and disposal equipment is available
  - Determine waste management needs and make a plan for proper disposal
  - Determine food needs
    - Ask about food and other allergies
  - Buy food
  - Buy or make sure that essential research equipment is available
  - Schedule pre-course meeting with teaching team
  - Schedule pre-course meeting with students
    - Introduce course expectations and guidelines
    - Personal check-list
    - Travelling check-list
    - Discuss accommodations
    - Discuss available infrastructure
    - Request information about food needs, allergies
    - Request relevant medical information
    - Provide relevant information on financial aid and course credits
    - Introduce relevant course content
- ***Develop interpersonal and communication skills***
  - Provide and refine communication agreement with students
  - Talk and decide on how to make decisions as a team
  - Communicate unacceptable behaviors (e.g., sexual or racial harassment, violent language)
  - Guide students through collaborative projects (e.g. rapid research projects, whole class research projects)
  - Guide students through cooperative activities (e.g. menu planning, clean ups, setting up camp, carpooling)
  - Provide spaces for informal engagement with other students and the teaching team (e.g. van rides, dinners, walks, etc)
- ***Prioritize inclusive pedagogy (see Table 1 on paper for definitions and examples)***
- Backwards design
- Scaffolding
- Peer-mentoring
- Iterative projects
- Inquiry-led learning
- Immersive learning
- Low-stakes projects
- Collaborative learning
- Structured community building
- Unstructured community building

2) **Resources**
a. **Sample group agreement template. Students can read these in small groups and make additions in blank spaces that are then proposed for group adoption.**

**Take responsibility for self-care** so you are mentally and physically prepared to learn. Speak up and seek help when needed.

**Create an inclusive space** for everyone to learn through our words and actions.

**Mutual respect,** respect each other and yourselves, let others speak, treat others how you would want to be treated, and make sure everyone is able to contribute.

**Be kind & communicate honestly, with kindness** and assume positive intent; we all make mistakes in our words.

**Be present and participate**, leave distractions (phones, airpods) at the door as much as possible.

b. **A short list of additional links and resources for syllabi development and five critical practices for field-based teaching**

**Syllabi**

Eslami M, Denaro K, Collins P, Sumarsono JM, Dennin M, Sato B. (2024). [How syllabi relate to outcomes in higher education: A study of syllabi learner-centeredness and grade inequities in STEM.](https://journals.physiology.org/doi/full/10.1152/advan.00119.2020) *PLoS One*. 2024 Apr 17;19(4):e0301331. https://doi.org/10.1152/advan.00119.2020

Ewell, S. N., Harvey, A., Clark, A., Maloney, M. E., Stevison, L. S., & Ballen, C. J. (2025). [Instructor recommendations for student learning strategies and metacognition: An analysis of undergraduate biology syllabi](https://doi.org/10.1002/tea.21996)). *Journal of Research in Science Teaching*, 62(4), 1132–1158. https://doi.org/10.1002/tea.21996

1) **Critical Practice: Build a course culture of well-being**

[UC Field Operations Safety manual](https://www.ucop.edu/safety-and-loss-prevention/_files/field-research-safety/uc-field-research-safety-manual.pdf)

[Wilderness first-aid](https://www.redcross.org/take-a-class/cpr/wilderness-sports?srsltid=AfmBOoohzua52rHtvcvMGsyqO4UbP6MN8C-7GtF9oljZQnjM-gi0c1hR) and [Mental Health first-aid UCSC](https://www.mentalhealthfirstaid.org/case-studies/university-california-santa-cruz-ucsc/)

[Field Inclusive: Resources (safety and accessibility)](https://www.fieldinclusive.org/resources/)

[Field Futures: Resources (safety and sexual harassment & violence prevention)](https://www.fieldfutures.org/resources)

2) **Critical Practice: Prepare logistics**

Field Inclusive checklists: https://www.fieldinclusive.org/resources/

Secure checklist examples

[Pace S, Fleischner TL, Weisberg S, Moline A. 2017. Saying Yes to Environmental Field Studies: Successful Operations. Nat Hist Inst.](https://www.zotero.org/google-docs/?ogwt2f)  https://naturalhistoryinstitute.org/saying-yes-to-environmental-field-studies-a-guide-to-proactive-successful-administration-and-operations/

3) **Critical Practice: Develop interpersonal and communication skills**

[CIRTL Network](https://cirtl.net/)

[UC Davis Principles of Community](https://excellence.ucdavis.edu/principles-community)

4) **Critical Practice: Prioritize inclusive pedagogy**

**Inclusive pedagogy resources**

Tanner, K. D. (2013). "[Structure matters: Twenty-one teaching strategies to promote student engagement and cultivate classroom equity.](https://doi.org/10.1187/cbe.13-06-0115) *CBE—Life Sciences Education, 12*(3), 322–331.

[CIRTL Network Inclusive Teaching Resources.](https://cirtl.net/inclusive-teaching/)

[Georgetown University Inclusive Pedagogy Toolkit.](https://cndls.georgetown.edu/resources/ip-toolkit/)

**Field-specific inclusive pedagogy resources**

Zavaleta, E. S., Beltran, R. S., & Borker, A. L. (2020). "How field courses propel inclusion and collective excellence How field courses propel inclusion and collective excellence." *Trends in Ecology & Evolution, 35*(11), 953–956. <https://doi.org/10.1016/j.tree.2020.08.005>

Emery, N. C., Bledsoe, E. K., Hasley, A. O., & Eaton, C. D. (2021). Cultivating inclusive instructional and research environments in ecology and evolutionary science cultivating inclusive instructional and research environments in ecology and evolutionary science. *Ecology and Evolution*, *11*(4), 1480-1491. <https://doi.org/10.1002/ece3.7062>

Byrne, L. B., Rauschert, E. S. J., Rodgers, V. L., Bowser, G., Casper, A., Dewsbury, B., Morales, N., Vance‐Chalcraft, H. D., & Weber, L. (2025). Diversifying Ecology Education for Everyone Through More Inclusive, Interdisciplinary, and Accessible Teaching. *The Bulletin of the Ecological Society of America*, e2233. <https://doi.org/10.1002/bes2.2233>

1. [5) ](https://doi.org/10.1002/bes2.2233)**Critical Practice: Provide context and content**

[Field Inclusive: Gear Libraries](https://www.fieldinclusive.org/wp-content/uploads/2025/02/FI_How-to-Set-Up-and-Manage-a-Gear-Exchange-Program.pdf)

Based on your location, we invite you to research and learn about Traditional Ecological Knowledge and history of place.

[Teaching and learning with historic places.](https://www.nps.gov/subjects/teachingwithhistoricplaces/index.htm#:~:text=Teaching%20&%20Learning%20with%20Historic%20Places,Find%20Lesson%20Plans)

Brooks, S. D., Sabzalian, L., Weiser-Nieto, R., & Springer, S. (2023). “[We should have held this in a circle”: White ignorance and answerability in outdoor education.](https://canvas.ucsc.edu/courses/87739/files/11543218?wrap=1) *The Journal of Environmental Education*, *54*(2), 114–131. <https://doi.org/10.1080/00958964.2023.2169897>

Greenall & Bailey (2022). [An Instructor’s Guide to Including Traditional Ecological Knowledge in the Undergraduate Biology Classroom. CBE—Life Sciences Education](https://canvas.ucsc.edu/courses/87739/files/11544112?wrap=1) 21:4 https://www.lifescied.org/doi/10.1187/cbe.21-12-0340

Moran Henn, P., Ostergren, D. M., & Nielsen, E. (2010/2011). “Integrating traditional ecological knowledge (TEK) into natural resource management.” *Park Science, 27*(3). [https://in.nau.edu/wp-content/uploads/sites/128/2018/08/NAU-315-ek.pdf](https://in.nau.edu/wp-content/uploads/sites/128/2018/08/NAU-315-ek.pdf?utm_source=chatgpt.com)

Anderson, M. Kat. (2005). Tending the Wild: Native American Knowledge and the Management of California’s Natural Resources. University of California Press.

Berkes, F. (2018). *Sacred Ecology* (4th ed.). Routledge.

Rizzo-Martinez, M. (2022). *We Are Not Animals: Indigenous Politics of Survival, Rebellion, and Reconstitution in Nineteenth-Century California.* University of Nebraska Press.

c. **Sample safety plan**

All visitors to UC Santa Cruz Younger Lagoon Reserve are encouraged to use a field safety plan. The University of California offers guidance and forms related to this process through the [UCOP Field Research Safety Center of Excellence](https://www.ucop.edu/safety-and-loss-prevention/environmental/program-resources/field-research-safety/index.html). Other institutions may have their own process.

A field safety plan serves as a tool to document travel hazard assessment, communication plan, emergency procedures, and required training. This plan should not only identify hazards but indicate how they will be addressed and mitigated to minimize the risk to participants.

This University of California Field Safety Plan has been partially pre-filled with information applicable to a trip to UC Santa Cruz Younger Lagoon Reserve. The form includes staff contacts, medical services directions, and other site specific information (Map is publicly available via this link: https://youngerlagoonreserve.ucsc.edu/species-maps-resources/maps.html). Visitors are encouraged to fill in their trip specific information, print copies, and review the plan with all members of their group.
